# Multimodal integration of sleep electroencephalogram, brain imaging, and cognitive assessments: approaches using noisy clinical data

**DOI:** 10.1093/sleep/zsad305

**Published:** 2023-11-29

**Authors:** Diego R Mazzotti

**Affiliations:** Division of Medical Informatics, Department of Internal Medicine, University of Kansas Medical Center, Kansas City, KS, USA; Division of Pulmonary Critical Care and Sleep Medicine, Department of Internal Medicine, University of Kansas Medical Center, Kansas City, KS, USA

Understanding the relationship between brain structure, function, and behavior is a challenge that many have attempted to undertake. It could help us pinpoint the role of specific brain regions and how they interact towards characterizing novel biomarkers for neurological and psychiatric conditions of public health relevance such as cognitive decline, Alzheimer’s disease and related dementias, Parkinson’s disease, and among others [1]. Electrophysiological assessments during sleep have been shown to capture important signatures of brain function and cognition [2, 3], and the sleep electroencephalogram (EEG) have been proposed as a tool to estimate brain health [4], a marker that has been associated with import age-related outcomes [5, 6]. However, the feasibility of robust investigations using prospectively collected, representative and comprehensive brain imaging data, including functional imaging, and sleep electrophysiological data, along with neuropsychological assessments that cover a wide range of cognitive functions is limited. Due to the high participant impact and cost, studies often include small sample sizes [7, 8] and lack in representation of underserved gender, sex, race, and ethnic minorities [9], minimizing the impact and generalizability of such important findings to these groups.

A possible approach that helps bridge this gap involves leveraging existing clinical data, collected over many years in sleep laboratories and imaging facilities and integrate with cognitive assessments data from neurological clinics. These “real world” data sources, despite being collected for clinical purposes and knowingly subjected to biases outside of the rigorous control of investigators prospectively collecting data [10, 11], have the potential to highlight important signals that might deserve further investigation in more controlled settings. Conventionally, the medical community has taken a more reductionist approach, trying to identify a single or small set of metrics that could better represent someone’s disease severity, despite the wealth of data collected as part of sleep electrophysiological assessments and imaging analyses; the apnea–hypopnea index being the most well-known example in sleep medicine, in the context of obstructive sleep apnea [12]. With the broader availability of clinical information systems, electronic health records, computing power, and recent advances in artificial intelligence [13], such task is more achievable. Moreover, statistical methods and study design considerations that help minimize some of the potential biases of using “messy” clinical data exist [13], which brings hope and enthusiasm to the scientific community to support data integration efforts towards utilizing these data, largely “untouched,” to support evidence generation. Specific approaches relevant to our sleep medicine community have been proposed [14, 15], and many efforts to improve representation and harmonization of clinical sleep data are underway [16].

One such investigation is featured in this issue of *SLEEP* [17], where Wei, Ganglberger, et al. describe an interesting approach to integrate brain magnetic resonance imaging (MRI), sleep EEG extracted from polysomnography (PSG) recordings, and cognitive assessments, with the goal to determine associations between brain structure, brain function during sleep and cognition. This study leveraged data across clinical information systems to identify a cohort of patients (*N* = 623) that underwent clinical, full-night PSGs, and clinical brain MRIs within 5 years of each other and extracted relevant sleep EEG physiological features as well as MRI regional volumetric measurements. In a subset of patients with available data (*N* = 160), the authors extracted cognitive data from the electronic health records, supporting the multimodal investigation of brain structure, function, and behavior.

One interesting approach taken by authors was to first propose a series of hypothesis-driven analyses, informed by prior literature. These included assessments of associations between sleep spindles/sigma power, thalamic volumes, and memory deficits; sleep slow wave/delta power, cortical volumes, and impaired cognition; overall associations between sleep macro and microstructure and specific brain region volumes and cognition; and whether combining sleep EEG and MRI-derived brain volumes would predict cognition better than each data modality alone. Several of these a priori associations have been confirmed, such as positive correlations between delta power volumes of frontal lobe regions and thalamus, as well as between sleep spindle density and thalamic and hypothalamic volumes. Some associations were not confirmed, such as structural region and REM sleep characteristics, although literature linking REM sleep and specific brain regions is scarce and controversial [18, 19]. When the authors combined a priori MRI and sleep EEG features, prediction of cognition scores improved. These initial findings were also modified by age, sex, and other disease groups (dementia, mild cognitive impairment, depression, cancer, diabetes, congestive heart failure, peripheral vascular disease, cerebral vascular disease, and Charlson comorbidity index), but generally supporting initial hypotheses. Moreover, and perhaps also as expected, brain imaging features were more relevant to differentiate patients with mild cognitive impairment or dementia as compared to controls than sleep EEG features.

Next, the authors used a hypothesis-free data-driven approach, to extend our understanding of how variation in sleep EEG, brain region volumes and cognition relate to each other, and whether it detect further signals beyond the hypothesized correlations. To minimize the sleep EEG feature space (776 initial features), input data was grouped into 24 distinct clusters explaining variability in different macro and microstructural sleep measurements. These were then assessed against clinical traits, as well as brain region volumes and cognition. Major associations included positive correlations between cognition and “delta and theta/alpha power in REM sleep” and “delta/fast power ratio in W+N1.” Positive correlations of “N2+N3 alpha and sigma power, and spindle activity” and age, thalamus and striatum volumes were also observed. Finally, the authors demonstrated that including all sleep EEG and MRI features (as opposed to a priori features used in hypothesis-driven analyses) substantially improved prediction of chronological age and cognition, as well as specific pairwise relationships between sleep EEG features and MRI-derived brain regions. These findings support the notion that there is much more information hidden in the data than what we have been hypothesizing. The authors also demonstrate in a robust way how machine learning and artificial intelligence can help us pinpoint the missing variance in these signals towards better characterizing such biological phenomena of interest.

Some important limitations of the study are worth mentioning, particularly as they relate to the settings in which data was collected (i.e. clinical vs. research), and their inherent biases such as lack of generalizability to the population, potential effect modification due to ascertainment biases for comorbidities (despite authors using chart review, which still may not fully represent a patient’s full health record), and timing between PSGs, MRIs, and cognitive assessments. Addressing these limitations in future studies with larger and more diverse samples will be essential, as this clinical sample is still racially and ethnically homogeneous (>84% white and > 88% non-Hispanic/Latino), and stratified analyses (by sex, age groups, and time difference between assessments) currently resulted in small sample sizes, likely subjected to lower statistical power. Despite these limitations, this proof-of-principle study brings enthusiasm about the possibilities of leveraging noisy clinical data towards enhancing our understanding. Enabling multi-site studies to help mitigate these limitations will be fundamental as we continue our quest towards understanding brain structure, function during sleep and behavior.
